# Lactate indices as predictors of in-hospital mortality or 90-day survival after admission to an intensive care unit in unselected critically ill patients

**DOI:** 10.1371/journal.pone.0229135

**Published:** 2020-03-09

**Authors:** Yusuke Hayashi, Hiroshi Endoh, Natuo Kamimura, Taro Tamakawa, Masakazu Nitta

**Affiliations:** 1 Advanced Emergency and Critical Care Center, Niigata University Medical & Dental Hospital, Niigata City, Niigata, Japan; 2 Department of Emergency & Critical Care Medicine, Niigata University Faculty of Medicine, Niigata City, Niigata, Japan; University of Florida, UNITED STATES

## Abstract

**Background:**

We performed an exclusive study to investigate the associations between a total of 23 lactate-related indices during the first 24h in an intensive care unit (ICU) and in-hospital mortality.

**Methods:**

Nine static and 14 dynamic lactate indices, including changes in lactate concentrations (Δ Lac) and slope (linear regression coefficient), were calculated from individual critically ill patient data extracted from the Multiparameter Intelligent Monitoring for Intensive Care (MIMIC) III database.

**Results:**

Data from a total of 781 ICU patients were extracted, consisted of 523 survivors and 258 non-survivors. The in-hospital mortality rate for this cohort was 33.0%. A multivariate logistic regression model identified maximal lactate concentration at 24h after ICU admission (max lactate at T24) as a significant predictor of in-hospital mortality (odds ratio = 1.431, 95% confidence interval [CI] = 1.278–1.604, *p*<0.001) after adjusting for predefined confounders (age, gender, sepsis, Elixhauser comorbidity score, mechanical ventilation, renal replacement therapy, vasopressors, ICU severity scores). Area under curve (AUC) for max lactate at T24 was larger (AUC = 0.776, 95% CI = 0.740–0.812) than other indices (p<0.001), comparable to an APACHE III score of 0.771. When combining max lactate at T24 with APACHE III, the AUC was increased to 0.815 (95% CI:0.783–0.847). The sensitivity, specificity, and positive and negative predictive values for the cut-off value of 3.05 mmol/L were 64.3%, 77.4%, 58.5%, and 81.5%, respectively.

Kaplan-Myer survival curves of the max lactate at T24 for 90-day survival after admission to ICU demonstrated a significant difference according to the cut-off value (*p*<0.001).

**Conclusions:**

These data indicate that the maximal arterial lactate concentration at T24 is a robust predictor of in-hospital mortality as well as 90-day survival in unselected ICU patients with predictive ability as comparable with APACHE III score.

## Introduction

An elevated blood lactate concentration may result from anaerobic metabolism caused by tissue hypoxia, accelerated aerobic glycolysis via the Na-K ATPase due to excess β-adrenergic stimulation, or impaired clearance from liver [1,2]. Because lactate concentrations are easily measured with a standard blood gas analyzer, numerous studies have demonstrated that elevated lactate concentrations or lactate changes are strongly associated with patient outcomes [3–6].

Several lactate indices related to kinetics have been developed and shown to be effective predictors of outcome in diverse cohorts of critically ill patients [5–6]. Dynamic indices based on serial lactate changes may be more informative than static ones. In particular, changes in lactate concentrations (Δ Lac) have been extensively studied [7–11]. However, adequate time intervals or optimal cut-off values for Δ Lac have varied considerably among studies [7–12].

Instead, the optimal time interval of measuring lactate for therapeutic guidance or prediction of outcome has been emphasized. For example, after an extensive review of studies on lactate indices, Vincent et al. [12] concluded that lactate changes should be recorded every 1-2h. Furthermore, the “Hour-1 bundle” in the recently updated Surviving Sepsis Campaign recommended that lactate should be remeasured within 2-4h when initial lactate is >2mmol/L [13].

We performed the present exclusive study to investigate the associations between a total of 9 static and 14 dynamic lactate indices during the first 24h of an intensive care unit (ICU) and in-hospital mortality in unselected ICU patients.

## Materials and methods

### Data sources

The medical information mart for intensive care (Multiparameter Intelligent Monitoring for Intensive Care III [MIMIC-III] ver1.4) is a large, and freely available database comprising deidentified health-related data associated with over 40,000 patients who stayed in 5 critical care units of the Beth Israel Deaconess Medical Center (Boston, MA) between 2001 and 2012 [14]. The use of the MIMIC-III database was approved for HE after certification of CITI program by Massachusetts Institute of Technology (No. 25459972). All data in the present study were extracted from the database using the Structured Query Language (SQL) scripts.

### Data extraction

#### Patient cohort

Eligibility criteria included first ICU admission, ICU stay >26h, age >16 years, and initial arterial lactate concentration > 2 mmol/L.

The SQL scripts were retrieved from the GitHub website (https://github.com/MIT-LCP/mimic-code) and used to calculate patient characteristics, baseline vital sign and laboratory data, Acute Physiology and Chronic Health Evaluation (APACHE) III score, Simplified Acute Physiology Score (SAPS) II, and Sequential Organ Failure Assessment (SOFA) score.

#### Lactate data

*Time and phase*. To extract lactate data from the MIMIC database, times and phases of arterial lactate measurements were arbitrarily defined as the following 3 times and 2 phases:

▪ T0: between 2h before and 2h after admission to ICU
▪ T12: between 10h and 14h after admission to ICU
▪ T24: between 22h and 26h after admission to ICU
▪ Phase 0-12h: between 2h before and 14h after admission to ICU
▪ Phase 0-24h: between 2h before and 26h after admission to ICU

A minimum of a single lactate value was needed for each time. A minimum of two lactate values was needed for each phase. Patients whose data did not satisfy the above requirements were excluded from this study.

*Lactate index*. Lactate index was calculated as follows:

Static Index

▪ Max lactate at T0 = maximum value of lactate in T0
▪ Max lactate at T0 ≥ 3, ≥ 4, ≥ 5, ≥ 6 mmol/L
▪ Max lactate at T12 = maximum value of lactate in T12
▪ Max lactate at T24 = maximum value of lactate in T24
▪ Max lactate 0-12h = maximum value of lactate in phase 0-12h
▪ Max lactate 0-24h = maximum value of lactate in phase 0-24h

Dynamic Index

▪ Δ Lac 0-12h (%) = 100 × (Max lactate at T0—minimum value of lactate at T12)/ Max lactate at T0
▪ Δ Lac 0-12h ≥30%, ≥20%, ≥10%
▪ Δ Lac CL 0-24h (%) = 100× (Max lactate at T0—minimum value of lactate at T24) / Max lactate at T0
▪ Δ Lac 0-24h ≥30%, ≥20%, ≥10%
▪ Δ Lac 12-24h (%) = 100 × (Max lactate at T12—minimum value of lactate at T24) / Max lactate at T12
▪ Δ Lac 12-24h ≥30%, ≥20%, ≥10%
▪ Lactate SL 0-12h = 12 x (linear regression coefficient of lactate in phase 0-12h)
▪ Lactate SL 0-24h = 24 x (linear regression coefficient of lactate in phase 0-24h)

Linear regression coefficients were calculated using the analyzing function of SQL.

### Study endpoints

The primary endpoint was to determine the effective lactate indices as predictors of in-hospital mortality. The secondary endpoint was to examine whether the effective lactate indices could predict 90-day survival after admission to the ICU.

### Statistical analyses

Descriptive statics were computed for all variables, and normal distribution was assessed using the Shapiro-Wilk test. Categorical variables were presented as number and percentage (%) and were compared using chi-squared test or Fisher’s exact test. Continuous variables were presented as mean ± standard deviation (SD) for variables with normal distribution or as median (interquartile range [IQR]) for variables without normal distribution and compared using either Student’s *t* test or the Mann-Whitney *U* test, respectively.

The associations between lactate index and in-hospital mortality were analyzed by univariate and multivariate logistic regression models. The goodness of fit of the model was assessed using the Hosmer-Lemeshow test. The significant lactate indices by multivariate logistic regression model were adjusted for predefined confounders: age, gender, sepsis (infection with organ dysfunction), Elixhauser comorbidity score [15], mechanical ventilation, renal replacement therapy, vasopressors, and ICU severity scores. Results of the logistic regression model were presented as odds ratios (OR) and 95% confidence intervals (CI).

The receiver operating characteristic (ROC) curve with the area under the curve (ROC-AUC) was plotted to evaluate the predictive ability of the lactate index for in-hospital mortality. Non-parametric DeLong test [16] was used to compare significant differences among ROC-AUCs. In addition, Youden method was used to calculate an optimal cut-off value. Sensitivity, specificity, positive predicting value (PPV), and negative predicting value (NPV) were calculated at the optimal cut-off value.

Kaplan-Meier estimation was used to obtain 90-day survival curves from ICU admission for the significant predictors of in-hospital mortality by a dichotomy of the determined cut-off value.

Two-sided *p* values <0.05 were considered significant. All analyses were performed using software Stata/SE package version 15.0 (StataCorp, College Station, TX, USA).

## Results

**Fig 1:**
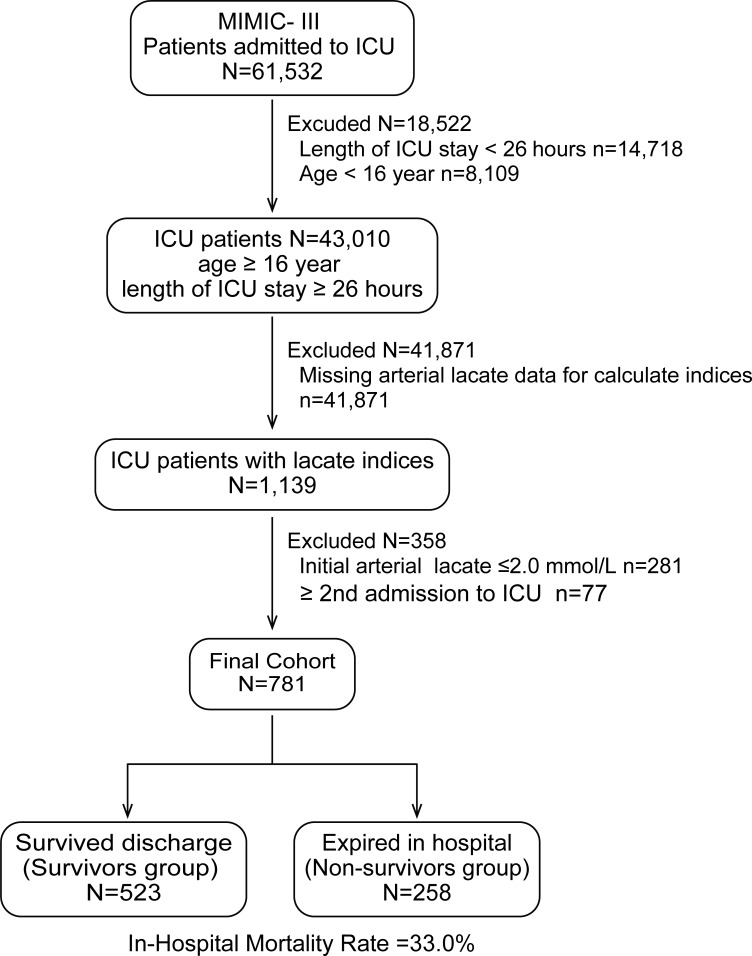
A flowchart of patient inclusion and exclusion.

Of the 61,532 patients admitted to 5 ICUs, 43,010 patients aged >16 years who stayed in the ICU for ≥26h were identified. After excluding the patients with missing lactate data (n = 41,871), initial arterial lactate ≤ 2.0 mmol/L (n = 281), and ≥ 2nd admission to ICU (n = 77), 781 patients were finally included for analysis and divided into a survived discharge group (survivors group: n = 523) and expired in hospital group (non-survivors group: n = 258). The in-hospital mortality rate for this cohort was 33.0% (Fig 1).

Baseline demographic characteristics for both groups are shown in Table 1.

**Table 1:** Baseline demographic characteristics.

Patients’ Characteristic	Total (n = 781)	Survivors (n = 523)	Non-survivors (n = 258)	*p* value
Age, year (range)	63 (51–75)	62 (49–74)	66 (54–78)	<0.001
Male, n (%)	465 (60.0)	320 (61.2)	145 (56.2)	0.182
Intensive Care Unit, n (%)				<0.001
Coronary care unit	52 (6.6)	24 (4.6)	28 (10.9)	
Medical intensive care unit	267 (34.2)	156 (29.8)	111 (43.0)	
Surgical intensive care unit	163 (20.9)	108 (20.7)	55 (21.3)	
Cardiac surgery recovery unit	139 (17.8)	118 (22.5)	21 (8.1)	
Trauma/surgical intensive care unit	160 (20.5)	117 (22.4)	43 (16.7)	
Primary international classification of disease version 9 diagnosis, n (%)				0.090
Neoplasms (140–239)	52 (6.7)	30(5.7)	22(8.5)	
Disease of nervous system (320–389)	181 (23.2)	108 (20.7)	73 (28.3)	
Diseases of the circulatory system (390–459)	185 (23.7)	130 (24.9)	55 (21.3)	
Disease of the respiratory system (460–519)	44 (5.6)	31 (5.9)	13 (5.0)	
Diseases of the digestive system (520–579)	100 (12.8)	62 (11.9)	38 (14.7)	
Injury and poisoning (800–999)	174 (22.3)	131 (20.1)	43 (16.7)	
Other	45 (5.2)	31(5.9)	14 (5.4)	
Sepsis	447 (57.2)	268 (51.2)	179 (69.4)	<0.001
Elixhauser comorbidity score	16 (7–27)	14 (5–24)	20 (11–30)	<0.001
Mechanical ventilation, n (%)	690 (88.4)	452 (86.4)	238 (92.3)	<0.05
Renal replacement therapy, n (%)	72 (9.2)	31 (5.9)	41 (15.9)	<0.001
Vasopressors, n (%)	623 (79.8)	384 (73.4)	239 (92.6)	<0.001
Length of ICU stay(day)	6.3 (3.1–13.1)	7.8 (3.9–13.8)	4.5 (2.3–10.7)	<0.001
Admission Type, n (%)				<0.001
Elective	103 (13.2)	85 (16.3)	18 (7.0)	
Emergency	666 (85.3)	432 (82.6)	234 (90.7)	
Urgent	12 (1.5)	6 (1.1)	6 (2.3)	
Ethnicity, n (%)				<0.05
White	541 (69.3)	370 (70.7)	171 (66.3)	
Black	78 (10.0)	56 (10.7)	22 (8.5)	
Asian	22 (2.8)	12 (2.3)	10 (3.9)	
Hispanic/Latino	26 (3.3)	19 (3.6)	7 (2.7)	
Others	114 (14.6)	66 (12.6)	48 (18.6)	
ICU Severity Score				
APACHE III	62 (46–84)	55 (42–71)	80 (65–100)	<0.001
SAPS II	49 (38–61)	43 (35–55)	60 (49–70)	<0.001
SOFA (1^st^ day)	9 (6–12)	8 (5–10)	11 (9–14)	<0.001

Values are expressed as median (IQR) or n (%).

Compared with survivors, non-survivors were older (*p*<0.001) and had a higher percentage of admission to a coronary care unit or medical intensive care unit, a higher percentage of sepsis (*p*<0.001), and a higher Elixhauser comorbidity score (*p*<0.001). Non-survivors also had a higher percentage of mechanical ventilation (1^st^ day) (*p*<0.05), a higher percentage of renal replacement therapy (1^st^ day) (*p*<0.001), a higher percentage of use of vasopressors (during ICU stay) (*p*<0.001), a shorter length of ICU stay (*p*<0.001), a higher percentage of emergency admission, a higher APACHE III score (*p*<0.001), SAPS II (*p*<0.001), and SOFA score (1^st^ day) (*p*<0.001). The distributions of primary international classification of disease version 9 diagnosis (ICD-9) did not differ between the groups (*p* = 0.090).

Baseline vital signs and laboratory data for both groups are shown in Table 2.

**Table 2:** Baseline vital signs and laboratory data.

Variable	Total (n = 781)	Survivors (n = 523)	Non-survivors (n = 258)	*p* value
Vital signs				
HR (beat/min)	95 (83–109)	94 (82–108)	98 (84–110)	<0.05
Systolic BP (mmHg)	109 (101–119)	110 (103–120)	107 (100–114)	<0.001
Diastolic BP (mmHg)	59 (53–65)	58 (53–66)	59 (51–65)	0.479
Respiratory Rate (breaths/min)	20 (17–24)	19 (17–22)	22 (19–26)	<0.001
Temperature (°C)	36.8 (36.3–37.3) n = 740)	36.9 (36.5–37.4) (n = 490)	36.6 (36.0–37.2) (n = 250)	<0.001
SpO_2_ (%)	98 (96–99)	98 (97–99)	97 (95–99)	<0.001
Laboratory Results				
Albumin (g/dL)	2.7 (2.3–3.2) (n = 511)	2.8 (2.4–3.3) (n = 310)	2.5 (2.1–3.0) (n = 201)	<0.001
Bicarbonate (mEq/L)	20.0 (17.2–22.8)	20.8 (18.3–23.5)	18.0 (15.3–21.0)	<0.001
Creatinine (mg/dL)	1.3 (0.9–2.1)	1.2 (0.9–1.8)	1.7 (1.1–2.6)	<0.001
Total bilirubin (mg/dL)	1.0 (0.5–2.0) (n = 623)	0.9 (0.5–1.6) (n = 393)	1.3 (0.9–2.1) (n = 230)	<0.001
BUN (mg/dL)	24.5 (16.4–38.5)	21.3 (14.7–34.2)	31.3 (20.0–47.0)	<0.001
Na (mEq/L)	138.8 (136.0–141.3)	138.8 (136.2–141.0)	138.7 (135.5–142.1)	0.972
Cl (mEq/L)	107.1 (103.3–110.7)	107.6 (104.0–111.8)	105.7 (101.2–111.6)	<0.001
K (mEq/L)	4.2 (3.9–4.5)	4.2 (3.8–4.5)	4.3 (3.9–4.7)	<0.01
Hb (g/dl)	10.6 (9.6–11.8)	10.6 (9.7–11.8)	10.5 (9.4–11.8)	0.199
HCT (%)	31.5 (28.4–34.8)	31.6 (28.6–34.3)	31.4 (28.2–35.3)	0.594
WBC(×10^3^/mm^3^)	12.5 (8.4–17.5)	12.6 (8.3–17.6)	12.5 (7.4–17.3)	0.237
Platelet (×10^3^/μL)	155 (107–235)	166 (121–239)	133 (93–214)	<0.001
Glucose (mg/dL)	144 (124–173)	142 (125–171)	146 (121–183)	0.204

Values are averaged within 24h of admission to ICU and expressed as median (IQR) or n (%).

Compared with survivors, non-survivors had a higher heart rate (*p*<0.05), a lower systolic blood pressure (*p*<0.001), a higher respiratory rate (*p*<0.001), a lower temperature (*p*<0.001), and a lower SpO_2_(*p*<0.001). Compared with survivors, non-survivors also had a lower albumin (*p*<0.001), a lower bicarbonate (*p*<0.001), a higher creatinine (*p*<0.001), a higher total bilirubin (*p*<0.001), a higher blood urea nitrogen (BUN) (*p*<0.001), a lower chloride (*p*<0.001), a higher potassium (*p*<0.01), and a lower platelet (*p*<0.001).

A total of 6,263 measurements of arterial lactate concentration were performed. The number of lactate measurements for each of the 3 time and the 2 phase categories are shown in S1 Table.

The comparisons of the 23 lactate indices between the groups are shown in Table 3.

**Table 3:** Comparisons of lactate index between survivors and Non-survivors groups.

Lactate index	Total (n = 781)	Survivors (n = 523)	Non-survivors (n = 258)	*p* value
Max lactate at T0 (mmol/L)	4.3 (3.0–6.6)	3.9 (2.9–5.7)	5.6 (3.5–9.1)	<0.001
Max lactate at T0 ≥3.0 (n) (%)	599 (76.7)	391 (74.8)	208 (80.6)	0.069
Max lactate at T0 ≥ 4.0 (n) (%)	438 (56.1)	260 (49.7)	178 (69.0)	<0.001
Max lactate at T0 ≥ 5.0 (n) (%)	316 (40.5)	176 (33.7)	140 (54.3)	<0.001
Max lactate at T0 ≥ 6.0 (n) (%)	238 (30.5)	119 (22.8)	119 (46.1)	<0.001
Max lactate at T12 (mmol/L)	3.2 (2.1–5.0)	2.8 (2.0–4.3)	4.3 (2.8–7.8)	<0.001
Max lactate at T24 (mmol/L)	2.4 (1.6–4.0)	2.0 (1.4–3.0)	4.2 (2.4–7.8)	<0.001
Max lactate 0-12h (mmol/L)	5.2 (3.8–7.9)	4.7 (3.5–6.9)	6.7 (4.6–10.5)	<0.001
Max lactate 0-24h (mmol/L)	5.4 (3.9–8.2)	4.9 (3.6–7.0)	7.3 (4.9–11.2)	<0.001
Lactate SL 0–12 (mmol/L/12h)	-0.748 (-2.138–0.501)	-0.919 (-2.088–0.182)	-0.432 (-2.368–1.389)	<0.01
Lactate SL 0-24h (mmol/L/24h)	-1.071 (-2.717–0.161)	-1.208 (-2.717–0.210)	-0.506 (-2.738–1.579)	<0.001
Δ Lac 0-12h (%)	29.2 (-1.8–52.3)	33.3 (6.7–54.0)	19.0 (-15.4–48.3)	<0.001
Δ Lac 0-24h (%)	44.4 (12.7–63.8)	50.0 (29.0–66.7)	19.6 (-20.3–51.8)	<0.001
Δ Lac 12-24h (%)	21.1 (-6.7–41.0)	25.0 (3.2–46.1)	7.4 (-21.7–31.5)	<0.001
Δ Lac 0-12h (≥30%) (n) (%)	386 (49.4)	282 (53.9)	104 (40.3)	<0.001
Δ Lac 0-12h (≥20%) (n) (%)	467 (59.8)	341 (65.2)	126 (48.8)	<0.001
Δ Lac 0-12h (≥10%) (n) (%)	535 (68.5)	376 (71.9)	159 (61.6)	<0.01
Δ Lac 0-24h (≥30%) (n) (%)	503 (64.4)	388 (74.2)	115 (44.6)	<0.001
Δ Lac 0-24h (≥20%) (n) (%)	550 (70.4)	422 (80.7)	128 (49.6)	<0.001
Δ Lac 0-24h (≥10%) (n) (%)	598 (76.6)	447 (85.5)	151 (58.5)	<0.001
Δ Lac 12-24h (≥30%) (n) (%)	300 (38.4)	234 (44.7)	66 (25.6)	<0.001
Δ Lac 12-24h (≥20%) (n) (%)	408 (52.2)	308 (58.9)	100 (38.8)	<0.001
Δ Lac 12-24h (≥10%) (n) (%)	487 (62.4)	366 (70.0)	121 (46.9)	<0.001

Values are expressed as median (IQR) or n (%).

All lactate indices other than max lactate at T0 (≥ 3mmol/L) were significantly different between the groups.

Results of univariate or multivariate logistic model assessments of the lactate indices for in-hospital mortality are shown in Table 4.

**Table 4:** Univariate and multivariate logistic regression models of lactate index for in-hospital mortality.

Lactate Index	Univariate Logistic Regression Model			Multivariate Logistic Regression Model		
	OR (95% CI)	Z value	*p* value	OR (95% CI)	Z value	*p* value
Max lactate at T0 (mmol/L)	1.200 (1.142–1.260)	7.25	<0.001	1.422 (1.065–1.898)	2.39	<0.05
Max lactate at T0 ≥3.0 (mmol/L)	1.404 (0.974–2.026)	1.82	0.069	0.627 (0.345–1.138)	-1.53	0.125
Max lactate at T0 ≥ 4.0 (mmol/L)	2.251 (1.643–3.083)	5.05	<0.001	1.620 (0.845–3.109)	1.45	0.147
Max lactate at T0 ≥ 5.0 (mmol/L)	2.239 (1.725–3.173)	5.46	<0.001	0.611 (0.290–1.287)	-1.30	0.195
Max lactate at T0 ≥ 6.0 (mmol/L)	2.906 (2.113–3.998)	6.56	<0.001	1.431 (0.656–3.121)	0.90	0.368
Max lactate at T12 (mmol/L)	1.264 (1.196–1.336)	8.30	<0.001	0.871 (0.660–1.149)	-0.98	0.329
Max lactate at T24 (mmol/L)	1.503 (1.399–1.627)	10.08	<0.001	1.399 (1.102–1.776)	2.76	<0.01
Max lactate 0-12h (mmol/L)	1.203 (1.149–1.259)	7.97	<0.001	1.322 (0.895–1.954)	1.40	0.161
Max lactate 0-24h (mmol/L)	1.226 (1.173–1.283)	8.92	<0.001	0.664 (0.465–0.948)	-2.25	<0.05
Lactate SL 0-12h (mmol/L/12hrs)	1.084 (1.028–1.144)	2.96	<0.01	0.977 (0.796–1.199)	-0.23	0.821
Lactate SL 0-24h (mmol/L/24hrs)	1.115 (1.062–1.171)	4.38	<0.001	1.040 (0.929–1.165)	0.68	0.497
Δ Lac 0-12h (%)	0.994 (0.991–0.997)	-4.22	<0.001	0.994 (0.984–1.004)	-1.20	0.230
Δ Lac 0-24h (%)	0.987 (0.983–0.990)	-7.77	<0.001	0.990 (0.978–1.001)	-1.74	0.082
Δ Lac 12-24h (%)	0.989 (0.985–0.993)	-5.82	<0.001	1.001 (0.993–1.008)	0.14	0.889
Δ Lac 0-12h (≥30%)	0.577 (0.427–0.781)	-3.56	<0.001	1.556 (0.764–3.169)	1.22	0.223
Δ Lac 0-12h (≥20%)	0.509 (0.376–0.690)	-4.36	<0.001	0.464 (0.205–1.053)	-1.84	0.066
Δ Lac 0-12h (≥10%)	0.628 (0.458–0.861)	-2.89	<0.01	2.349 (1.138–4.850)	2.31	<0.05
Δ Lac 0-24h (≥30%)	0.280 (0.204–0.383)	-7.95	<0.001	0.924 (0.399–2.136)	-0.19	0.852
Δ Lac 0-24h (≥20%)	0.236 (0.170–0.327)	-8.67	<0.001	0.674 (0.250–1.818)	-0.78	0.435
Δ Lac 0-24h (≥10%)	0.240 (0.170–0.340)	-8.06	<0.001	1.170 (0.502–2.729)	0.36	0.716
Δ Lac 12-24h (≥30%)	0.425 (0.306–0.590)	-5.11	<0.001	0.959 (0.521–1.765)	-0.13	0.893
Δ Lac 12-24h (≥20%)	0.442 (0.326–0.599)	-5.25	<0.001	1.282 (0.619–2.653)	0.67	0.504
Δ Lac 12-24h (≥10%)	0.379 (0.278–0.515)	-6.18	<0.001	0.863 (0.438–1.701)	-0.43	0.671

Hosmer-Lemeshow goodness-of-fit test (*p* value = 0.529).

Multivariate logistic regression model identified max lactate at T0 (*p*<0.05), max lactate at T24 (*p*<0.01), max lactate 0-24h (*p*<0.05), and ΔLacl 0-12h (≥10%) (*p*<0.05) as significant predictors for in-hospital mortality. Hosmer-Lemeshow test was not significant (*p* = 0.529) indicating that the model adequately fit to data.

Of the 4 predictors, max lactate at T24 remained a significant predictor of in-hospital mortality after adjusting for predefined confounders (OR = 1.431, 95% CI = 1.277–1.604, *p*<0.001) (Table 5). Furthermore, APACHE III score and age (year) remained as significant predictors (Table 5).

**Table 5:** Multivariate logistic regression model and ROC-AUC for predictor of in-hospital mortality.

Predictor	Multivariate Logistic Regression Model			ROC-AUC
	OR (95% CI)	Z value	*p* value	(95% CI)
Age (year)	1.017 (1.003–1.032)	2.31	<0.05	0.574 (0.531–0.617) ^¶^^,^ ^†^
Gender(male = 1)	0.897 (0.612–1.316)	-0.55	0.580	0.475 (0.438–0.512) ^¶^^,^ ^†^
Sepsis	0.919 (0.601–1.404)	-0.39	0.695	0.591 (0.555–0.626) ^¶^^,^ ^†^
Elixhauser comorbidity score	1.014 (0.998–1.030)	1.73	0.084	0.628 (0.587–0.669) ^¶^^,^ ^†^
Mechanical ventilation	1.520 (0.557–2.939)	1.25	0.213	0.529 (0.507–0.551) ^¶^^,^ ^†^
Renal replacement therapy	1.070 (0.581–2.057)	0.20	0.840	0.550 (0.525–0.574) ^¶^^,^ ^†^
Vasopressors	1.549 (0.839–2.859)	1.40	0.161	0.596 (0.571–0.621) ^¶^^,^ ^†^
APACHE III	1.016 (1.002–1.030)	2.27	<0.05	0.771 (0.737–0.805)
SAPS II	1.010 (0.985–1.036)	0.81	0.419	0.753 (0.717–0.789)
SOFA(1^st^ day)	1.042 (0.965–1.124)	1.05	0.295	0.738 (0.700–0.777)
Max lactate at T0 (mmol/L)	1.154 (0.996–1.338)	1.91	0.056	0.639 (0.596–0.682) ^¶^^,^ ^†^
Max lactate at T24 (mmol/L)	1.434 (1.299–1.629)	6.49	<0.001	0.776 (0.740–0.812)
Max lactate 0-24h (mmol/L)	0.882 (0.760–1.023)	-1.66	0.096	0.699 (0.659–0.739) ^†^^,^ ^ǂ^
Δ Lac 0-12h (≥10%)	1.316 (0.796–2.176)	1.07	0.284	0.449 (0.413–0.484) ^¶^^,^ ^†^

Hosmer-Lemeshow goodness-of-fit test (*p* value = 0.713).

¶ and † indicate statistically different from APACHE III and Max lactate at T24, respectively (*p*<0.001 for both).

ǂ indicates statistically different from Max lactate at T24 (*p*<0.01).

The ROC-AUC of max lactate at T24 (AUC = 0.776, 95%CI = 0.740–0.812) was larger than other lactate indices (Table 5). When combining max lactate at T24 with APACHE III, the AUC was increased to 0.815 (95% CI:0.783–0.847) (S1 Fig).

The optimal cut-off values, Youden index, sensitivity, specificity, PPV, and NPV for max lactate at T24, APACHE III score, and age for in-hospital mortality are shown in Table 6.

**Table 6:** Cut-off value, Youden index, sensitivity, specificity, PPV, and NPV of predictors of in-hospital mortality.

Predictor	Cut-off Value	Youden Index	Sensitivity (%) (95% CI)	Specificity (%) (95% CI)	PPV (%) (95% CI)	NPV (%) (95% CI)
Max lactate at T24 (mmol/L)	3.05	0.418	64.3 (58.2–70.2)	77.4 (73.6–81.0)	58.5 (52.5–64.2)	81.5 (77.8–84.8)
Age (year)	76.5	0.111	30.2 (24.7–36.2)	81.5 (77.9–84.7)	44.6 (37.1–52.3)	70.3 (66.5–73.9)
APACHE III	64.5	0.423	75.2 (69.5–80.3)	67.1 (62.9–71.1)	53.0 (47.7–58.2)	84.6 (80.7–87.9)

**Fig 2:**
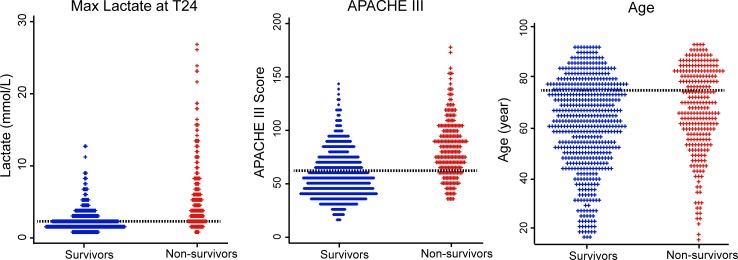
Dot plot for distribution of max lactate at 24h, APACHE III score, and age between survivors (n = 523) and non-survivors (n = 258). The dotted lines indicate cut-off values.

The distributions of individual values of max lactate at T24, APACHE III score, and age between survivor and non-survivors are shown in Fig 2.

**Fig 3:**
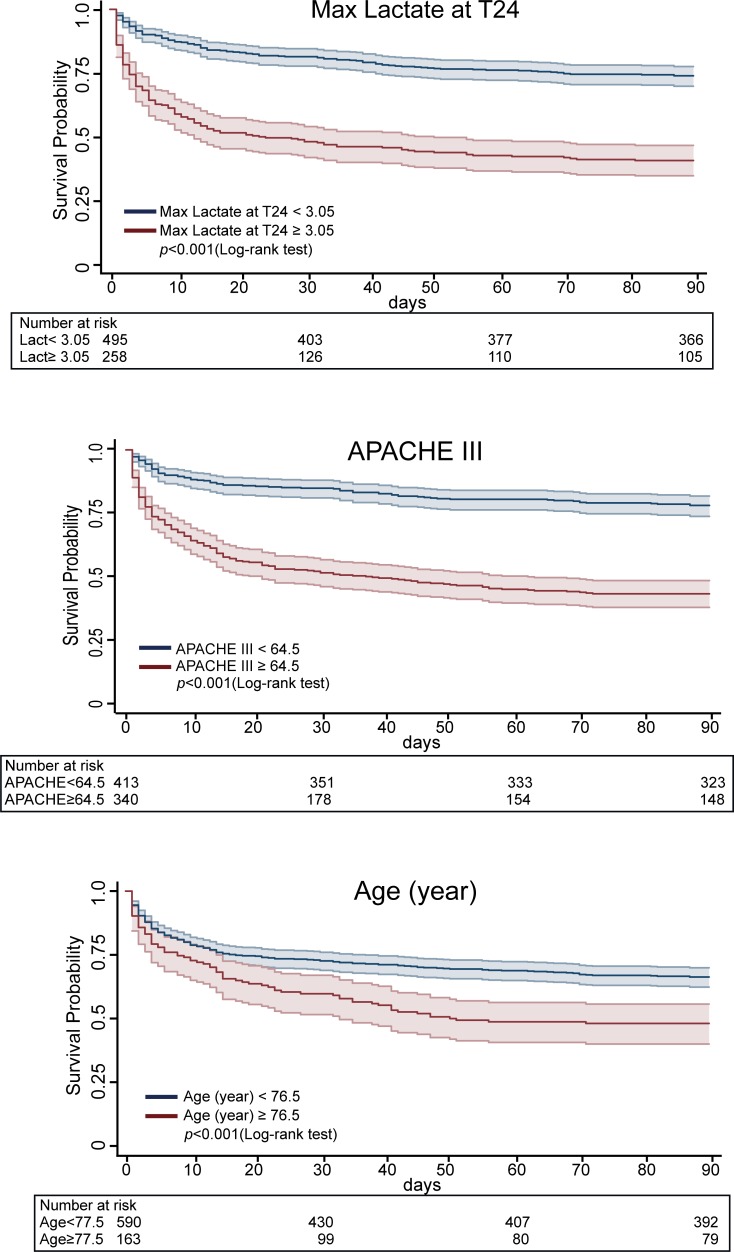
Kaplan-Meier survival curve with risk tables and 95% confidence intervals of Max lactate at T24, APCHE III score, and age for 90-day survival after ICU admission.

Kaplan-Meier survival curves with risk tables and 95% confidence intervals of the max lactate at T24, APACHE III score, and age for 90-day survival after admission to ICU according to the cut-off values are shown in Fig 3.

There was a significant difference between survival curves using the cut-off values (*p*<0.001 for all).

## Discussion

In the present study of unselected ICU adult patients extracted from the MIMIC III database, we have shown that max lactate at T24 was a significant predictor for both in-hospital (OR = 1.431, 95% CI = 1.277–1.604, *p*<0.001) and 90-day survival. The ROC-AUC of max lactate at T24 (AUC = 0.776, 95%CI = 0.740–0.812) was larger than other indices, as comparable with APACHE III score.

### MIMIC database and lactate study

Many studies have used the MIMIC II or MIMIC III database. According to the MIMIC website (https://mimic.physionet.org/about/publications/), a total of 145 studies have been published between 2004 and 2018. However, as far as we know, few studies have focused on lactate dynamics. Moskowitz et al. [17] showed that hypo-magnesium (<1.6 mg/dL) was associated with an increased risk of mild (>2 mmol/L) and severe (>4 mmol/L) lactic acidosis in 8,922 critically ill patients extracted from the MIMIC II database. Using a fractional polynomial Cox-proportional hazard model, Zhang et al. [18] reported that higher initial lactate concentration and longer lactate normalization time were significantly associated with increased hazard of mortality in 6,291 ICU patients from the MIMIC II database. Recently, Liu et al. [19] described that the first 24h averaged lactate value was an independent prognostic factor of mortality and the discriminative power of which was superior to qSOFA and comparable to SOFA in 1865 septic patients extracted from MIMIC III. However, dynamic lactate index was not evaluated in these studies.

### Static lactate index

Several studies have indicated that a single initial lactate concentration in prehospital, emergency department (ED), or ICU settings is a strong predictor of outcome [20–23]. Martín-Rodríguez et al. [20] demonstrated an efficient cut-off value of prehospital lactate ≥4.25 mmol/L for 30-day mortality in 279 emergency patients. Similarly, Jansen et al. [21] described that prehospital lactate ≥4.0 mmol/L was highly associated with in-hospital mortality, providing more prognostic information than systolic blood pressure or heart rate in 124 patients. Chebl et al. [22] also reported that lactate >4.0 mmol/L in the ED was associated with an increased adjusted OR (>29.0) for in-hospital mortality. Trzeciak et al. [23] reported a strong association between initial lactate ≥ 4.0 mmol/L, wherever measured (ED, ICU, or general ward), and acute-phase death (≤ 3 days) in infectious patients. In this study, initial maximal lactate (max lactate at T0) was barely not statistically significant after adjusting for confounders (*p* = 0.056) (Table 5).

Recently, Masyuk et al. [24] showed a dynamic index, Δ 24Lac (the difference between maximum lactate on day 1 and day 2) was associated with in-hospital and long-term mortality in unselected ICU patients. The predictor of max lactate at T24 in this study is the final concentration of lactate over the first 24h, which may be relevant to the Δ 24 Lac.

### Dynamic lactate index

Several dynamic lactate indices have been shown as effective predictors of outcome in ICU patients. ΔLac, also called lactate clearance in some articles, has been extensively studied [7–11]. However, use of the term “clearance” has been criticized because of its general meaning of elimination and the need for intravenous injection of radiolabeled lactate to directly measure clearance [2,12]. Therefore, this article uses the term ΔLac rather than clearance when describing other studies that did not use radiolabeled lactate to directly measure clearance. Nguyen et al. [7] showed that a lower ΔLac 0-6h, obtained at 0h and 6h after admission to ED, was significantly associated with in-hospital mortality in 111 adult patients with severe sepsis or septic shock. Similarly, Ryoo et al. [8] reported that median 6h repeated lactate concentrations and ΔLac 0-6h were significantly associated with 28-day mortality in 1,060 adult patients with septic shock. In contrast, the raw data for ΔLac varied considerably, resulting in a lower diagnostic ability [7, 8]. However, in the systematic review and meta-analysis for ΔLac, Zang and Xui [11] concluded that the diagnostic performance of ΔLac remained at a moderate accuracy level in the heterogenous ICU population. In our study, none of the 12 lactate indices related ΔLac was identified as a significant predictor. A possible explanation may be that ΔLac was calculated based on maximum and minimum values, leading to exaggerated variations.

In contrast, slope (SL; linear regression coefficient) may more accurately reflect changes of lactate concentrations than ΔLac. Actually, Nichol et al. [25] calculated lactate SL 0-24h in 5,041 unselected critically ill patients and demonstrated that lactate SL 0-24h and time-weighted lactate concentration over the first 24h ICU stay were highly significant predictors of in-hospital mortality. Their median value of lactate SL 0-24h ranged from -0.30 for survivors to -0.21 mmol/L/24h for non-survivors after eliminating extreme outliers; these values are approximately 3–4 times smaller than values in our study (Table 3), probably attributable to the difference in mortality rates (19.2% vs. 33.0%) and the presence of outliers. Nicolet al. [25] also reported that OR for hospital mortality was significantly greater in patients with slopes >0 or 0 to -1 compared with patients with a slope <-1. In our study, lactate SL 0-24h was not a significant predictor of in-hospital mortality. Interestingly, OR for hospital mortality was similarly shown to be greater in patients with lactate SL 0-24h ≥0 (n = 219) or 0 > lactate SL 0-24h ≥ -1 (n = 159) compared with -1 > lactate SL 0-24h (n = 403) after adjusting for the predefined confounders (OR = 1.557, CI = 1.040–2.332, *p*<0.05, and OR = 1.042, CI = 0.648–1.674, *p* = 0.866, respectively).

### Limitations

The present study has several limitations. First, this study was based on a single institutional database. Second, due to missing lactate data, the final cohort resulted in a small fraction of the overall MIMIC III data (781/61,532 = 1.27%) that may not reflect the entire MIMIC III data. Third, times for lactate measurements were not precisely protocoled due to the retrospective nature of the study, resulting in broader measuring times for calculating lactate indices. Fourth, calculation of ΔLac was based on maximal and minimum values of lactate concentrations, leading to exaggerated results. Fifth, although results were adjusted for confounders including ICU severity score, more serious patients might have more lactate measurements, which could affect values of static or dynamic index. Finally, the treatments against elevated lactate concentrations were not protocoled or uniformly reported due to the retrospective nature of the study and heterogenous cohorts.

## Conclusions

Among 23 lactate-related indices during the first 24h following ICU admission, a peak value of arterial lactate concentration at 24h was a robust independent predictor of in-hospital mortality in unselected critically ill patients extracted from the MIMIC III database. In addition, the peak arterial lactate concentration at 24h could also predict 90-day survival after admission to ICU.

## Supporting information

S1 Fig Receiver operating curves for max lactate at 24h, APACHE III, and max lactate at 24h + APACHE III. (EPS)

S1 Table Numbers of lactate measurements in times and phases. (DOCX)

S1 Data (XLS)

## List of abbreviations

APACHE: Acute Physiologic and Chronic Health Evaluation
CI: confidence interval
ΔLac: changes in lactate concentrations
ED: emergency department
ICU: intensive care unit
IQR: interquartile range
MIMIC: medical information mart for intensive care
NPV: negative predictive value
OR: odds ratio
PPV: positive predictive value
ROC: receiver operating characteristic
ROC-AUC: area under the receiver operating characteristic curve
SAPS: Severity Acute Physiologic Score
SD: standard deviation
SL: slope
SQL: Structured Query Language
SOFA: Sequential Organ Failure Assessment
