# Supplementary material for: Lactate indices as predictors of in-hospital mortality or 90-day survival after admission to an intensive care unit in unselected critically ill patients
Source: PLoS One. 2020 Mar 9;15(3):e0229135. doi: 10.1371/journal.pone.0229135 (PMC7062275; doi:10.1371/journal.pone.0229135)
Supplement: S1 Table — (DOCX) [file pone.0229135.s002.docx]

S1 Table. Numbers of Lactate Measurements in Times and Phases

| Time / Phase | Total (n=781) | Survivors (n=523) | Non- survivors (n=258) |
| --- | --- | --- | --- |
| T0 | 2 (1-3)  Range:1-8 | 2 (1-3)  Range:1-7 | 2 (1-3)  Range:1-8 |
| T12 | 1 (1-2)  Range:1-4 | 1 (1-2)  Range:1-4 | 1 (1-2)  Range:1-4 |
| T24 | 1 (1-1)  Range:1-4 | 1 (1-1) ^¶^  Range:1-4 | 1 (1-1)  Range:1-4 |
| Phase 0-12h | 5 (4-7)  Range:2-18 | 5 (4-7)  Range:2-18 | 5 (4-7)  Range:2-13 |
| Phase 0-24h | 7 (6-10)  Range:2-20 | 7 (6-9) ^†^  Range:2-20 | 8 (6-11)  Range:3-20 |

Values are expressed as median (IQR) and range per patient.

¶ and † indicate statistically different from non-survivor group (*p*<0.001 and *p*<0.01, respectively).
